# P01 - Sensitisation pattern to inhalant allergens in Armenian children

**DOI:** 10.1186/2045-7022-4-S1-P56

**Published:** 2014-03-14

**Authors:** Astghik Baghdasaryan, Ashot Sarkissian, Ernst Leumann, Roger Lauener

**Affiliations:** 1 “Arabkir” Joint Medical Centre-Institute of Child and Adolescent Health (“Arabkir” JMC-ICAH), Yerevan, Armenia; 2 Zurich University Children’s Hospital, Zurich, Switzerland

## Background

Pediatric respiratory allergies are increasing problem in Armenia being underestimated according to official reports. “Allergic Sensitization and Diseases in Armenian Children” study has been conducted to assess the prevailing sensitizations to inhalant allergens of Armenia using the standardized panel and method of PEP study (Pan-European standard Skin prick test study) conducted by Global Allergy and Asthma European Network (GA2LEN).

## Objectives

To reveal prevailing sensitizations to inhalant allergens in Armenian children presenting atopy with a standardized method for diagnosis (developed by GA2LEN for European Centers), and to compare the obtained data with participant countries of PEP project.

## Materials and methods

A total of 231 children aged 2-18 years applied to “Arabkir” MC with previous history or suspicion of atopy were evaluated for sensitization to inhalant allergens using standardized prick test method, allergen solutions and panel. Additional allergen (Poplar) was used for Armenia. Data were saved and analyzed in SPSS software.

## Results

192 (83%) of all investigated children had sensitization to at least 1 allergen. 31 (13% of all) children had monosensitization, 161 (70%) had polysensitization up to maximum 12 allergens. The most prevalent allergen in Armenia, as in Europe, was the grass mix: 115 (49.8%). In comparison to European countries, where Birch pollen was the 3^rd^ important allergen, tree pollen allergens were less important for Armenia: the most prevalent one was the plane 13.4% (31). 10 allergens allowed identification of more than 95% of sensitized subjects (grass mix, *Dermatophagoides pteronyssinus*, dog, *Alternaria*, Plane, *Artemisia*, Hazel/Olive (or Ash), Cat/ *Dermatophagoides farinae*). 12 allergens were needed to identify all sensitized children (grass mix, *Dermatophagoides pteronyssinus*, dog, *Alternaria*, Plane, *Artemisia*, Hazel/Olive (or Ash), Cat/*Dermatophagoides farinae*, *Cladosporium/* Poplar)*.*

## Conclusions

The most important inhalant allergen in Armenia was grass pollen. 10 allergens allowed the identification of the majority of sensitized children.

